# Analysis of neuronal cardiolipin and monolysocardiolipin from biological samples with cyclic ion mobility mass spectrometry

**DOI:** 10.3389/fphys.2025.1592008

**Published:** 2025-05-29

**Authors:** Katlynn J. Emaus, Carmen A. Dunbar, Joseph Caruso, Brandon T. Ruotolo, Joseph M. Wider, Thomas H. Sanderson

**Affiliations:** ^1^ Neuroscience Graduate Program, University of Michigan, Ann Arbor, MI, United States; ^2^ Department of Emergency Medicine, University of Michigan, Ann Arbor, MI, United States; ^3^ University of Michigan Biological Mass Spectrometry Core Facility, Ann Arbor, MI, United States; ^4^ Department of Chemistry, University of Michigan, Ann Arbor, MI, United States; ^5^ Olink, Thermo Fisher Scientific, Waltham, MA, United States; ^6^ The Max Harry Weil Institute for Critical Care Research and Innovation, University of Michigan, Ann Arbor, MI, United States; ^7^ Department of Molecular and Integrative Physiology, University of Michigan, Ann Arbor, MI, United States

**Keywords:** cardiolipin, monolysocardiolipin, mass spectrometry, cyclic ion mobility mass spectrometry, clinical application

## Abstract

The mitochondrial phospholipid cardiolipin (CL) is essential for proper mitochondrial function and energy production. Cardiolipin has four distinct fatty acid tails with varying expression compositions, resulting in a highly variable tissue-specific distribution of isomer expression. Neuronal cardiolipin has a remarkable variety of subspecies and has recently been used as a biomarker to predict brain injury severity following cardiac arrest and traumatic brain injury. Multiple conditions have been associated with disordered cardiolipin remodeling, including Alzheimer’s disease, Parkinson’s disease, Barth syndrome, and astrocytoma. The clinical relevance of cardiolipin as a biomarker and the importance of the mechanistic role of cardiolipin remodeling in disease emphasize the demand for a reliable and accurate means of the identification and quantification of cardiolipin. In this study, we outline the use of a novel method of cardiolipin analysis using cyclic ion mobility mass spectrometry (cIMS-MS) to isolate and identify cardiolipin subspecies in several biological samples. Furthermore, cIMS-MS established the composition of the cardiolipin profile by individual subspecies across biological samples under basal conditions. Monolysocardiolipin (MLCL), the precursor of mature cardiolipin and a primary diagnostic biomarker of Barth syndrome, was isolated from cardiolipin and identified. The monolysocardiolipin:cardiolipin ratio was quantified in brain samples from tafazzin-knockout (KO) mice, demonstrating accumulation of MLCL and providing direct evidence for the validity of this cIMS-MS methodology through genetic loss-of-function. The novel, multiple-pass feature of cIMS-MS enabled the isolation and amplification of less abundant cardiolipin subspecies in both standards and biological samples. This protocol enables rapid analysis of biological samples, allowing researchers to further dissect the mechanistic role of cardiolipin in injury pathology, with simplified sample preparation and reduced potential for artifact introduction.

## Introduction

Cardiolipin (CL) is an essential phospholipid found almost exclusively in the inner membrane of the mitochondria, where it is a critical contributor to mitochondrial dynamics, cellular respiration, and overall cell health. As an essential structural and functional component in mitochondria, cardiolipin has been implicated in various aspects of health and disease. Brain-specific cardiolipin has been identified as a predictive biomarker for pediatric traumatic brain injury in rodents (Anthonymuthu et al., 2019a). In addition, neuronal-specific cardiolipin detected in serum following cardiac arrest in adults has been used to predict the severity of brain injury in a time-dependent manner (Anthonymuthu et al., 2019b). Accordingly, there has been considerable interest in the use of cardiolipin as a biomarker and as a potential predictor of injury severity. In astrocytoma tumor samples, long-chain cardiolipin species were significantly less abundant than those in healthy counterparts (Krieger et al., 2023). Abnormal cardiolipin profiles have been found in several neurodegenerative disorders, including Alzheimer’s disease and Parkinson’s disease, and in the early pathology of hypertension and type II diabetes (Falabella et al., 2021; Hao et al., 2024). In addition to its potential role in disease and trauma, disrupted cardiolipin remodeling is the hallmark of Barth syndrome, a metabolic disorder that affects the heart, muscle development, and cognitive function (Ikon and Ryan, 2017).

Cardiolipin is synthesized in the mitochondria and comprises two phosphatidyl moieties and four fatty acyl chains bound by a glycerol. Postsynthetic modifications of the acyl chain composition allow high variability in the structural species, which has been demonstrated to be tissue-specific (Oemer et al., 2020). Remodeling occurs when premature cardiolipin undergoes de-acylation of saturated acyl chains, producing the monolysocardiolipin (MLCL) intermediate (Neto et al., 2024). Monolysocardiolipin is then re-acylated with an unsaturated acyl chain by the enzyme tafazzin, producing a more stable mature cardiolipin. Tafazzin disruption that occurs in Barth syndrome is associated with an accumulation of monolysocardiolipin, leading to fatal cardiomyopathy, stunted growth, and cognitive and behavioral deficits.

Rigorous and reliable analysis of cardiolipin composition is essential in understanding the role of its biosynthesis, remodeling, and expression in disease and injury pathologies. Cardiolipin detection and analysis can be challenging due to tissue specificity and the variety of subspecies and isomers. Analysis is also complicated by more abundant phospholipids such as phosphatidylglycerol (PG), phosphatidylcholine (PC), and phosphatidylethanolamine (PE) that may mask the cardiolipin signal. Furthermore, the ability of separating cardiolipin from its precursor, monolysocardiolipin, relies on high resolution and degree of separation.

Liquid chromatography mass spectrometry (LC-MS) is one of the most widely used and accepted methods for cardiolipin analysis because the liquid chromatography (LC) step separates cardiolipin from other phospholipids, allowing for quantification of cardiolipin levels. For higher specificity, fragmentation can be used to target the intact lipids and then quantify the fragments produced, a process called tandem mass spectrometry (MS/MS), which has become a popular diagnostic tool in clinical settings (Thomas et al., 2022). Recently, there have been several reviews and methods published on optimizing protocols for cardiolipin identification and quantification (Amoscato et al., 2014; Bautista et al., 2022; Hsu et al., 2005; Lopalco et al., 2017; Minkler and Hoppel, 2010). Although these protocols have advanced the analysis of cardiolipin subspecies, there are some limitations to these methodologies (Bonner and Hopfgartner, 2022; Strasser and Váradi, 2000; Verpoorte et al., 2022). LC-MS/MS analysis has drawbacks, including the risk of introducing artifacts during LC, the development and validation of LC-MS/MS methods for clinical settings, costs associated with columns, standards, and training, and the lengthy sample preparation and run time.

**FIGURE 1:**
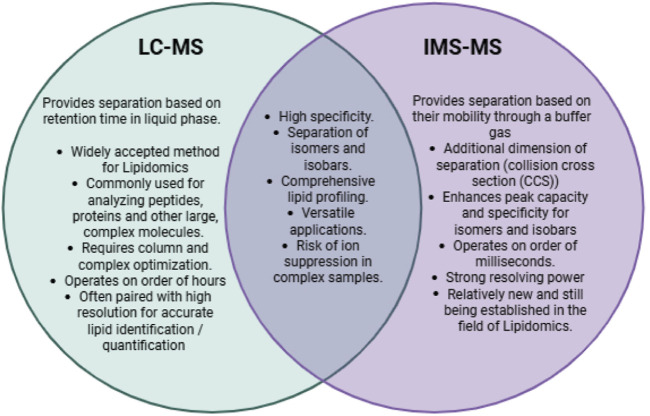
Comparison of LC-MS and traditional IMS-MS within lipidomics applications.

Another mass spectrometry technique that has become recognized as a powerful method for analyzing lipids is ion mobility spectrometry to mass spectrometry (IMS-MS). Due to the structural and chemical similarities of cardiolipin subspecies, achieving separation and resolution is critical for proper analysis. The combination of IMS and MS has proved to be integral in advancing mass spectrometry analysis by increasing the sensitivity and specificity of identification and quantification (Dodds and Baker, 2019). This technique has benefits over traditional LC-MS/MS, providing an additional layer of separation that can improve the resolution while decreasing the run time. The separation achieved by IMS occurs on the order of milliseconds, while LC separation requires minutes to hours. Additionally, the barrier to entry with IMS-MS is lower than that of LC-MS/MS, with less risk of artifact introduction, simplified troubleshooting when optimizing protocols, and avoiding the need for column separation prior to MS. Both are widely used for a variety of applications in lipidomics, both with their own advantages and limitations (Figure 1).

**FIGURE 2:**
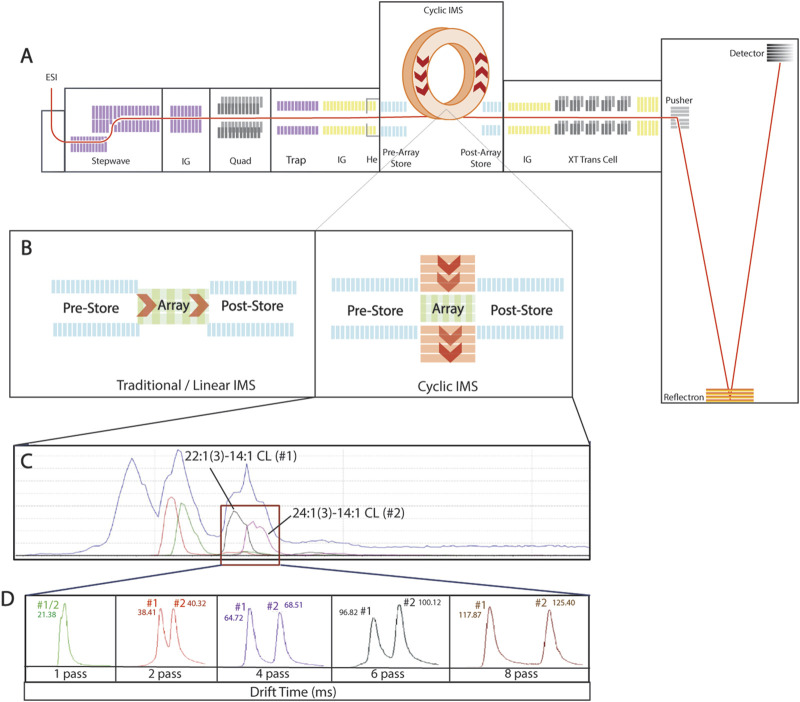
Multiple pass in cardiolipin synthetic standards. **(A)** Schematic representation of cyclic ion mobility mass spectrometry, highlighting the circular “racetrack” and allowing samples to achieve multiple passes and extending separation through the length of pass. **(B)** Direct comparison of traditional linear ion mobility mass spectrometry and cyclic ion mobility mass spectrometry. **(C)** Continuous live chromatogram shows the four cardiolipin subspecies in the cardiolipin internal standard mix [14:1 (3)–15:1 CL, 15:0 (3)–16:1 CL, 22:1 (3)–14:1 CL, and 24:1 (3)–14:1 CL]; the two subspecies intended for multiple passes [22:1 (3)–14:1 CL and 24:1 (3)–14:1 CL] are highlighted. **(D)** Chromatogram of 22:1 (3)–14:1 CL (#1) and 24:1 (3)–14:1 CL (#2) after one, two, four, six, and eight passes, demonstrating the novel ability to separate similar cardiolipin subspecies.

This study will outline the use of the cyclic ion mobility mass spectrometry (cIMS-MS) in assessing neuronal-specific cardiolipin subspecies from a variety of biological samples. cIMS-MS builds on the basis of traditional IMS-MS, but with the addition of a circular closed-loop traveling wave-enabled ion mobility separator (Giles et al., 2019). Traveling wave ion mobility (TWIM) uses stacked-ring ion guides and voltage currents to create an electric field that move the ions in waves through the separator. The resolution of traveling wave ion mobility spectrometry–mass spectrometry (TWIMS-MS) can be calculated as follows (Equation 1):.

$$R\;∼\;\sqrt{\left(LEQ\right)/\left(T\right),}$$ (1)

where R stands for resolution, L for the length of the path, E for applied electrical field, Q for charge of ion, and T for temperature. The addition of a circular TWIM extends the length of path (L) indefinitely and increases the resolution significantly. With each pass around the “racetrack,” the ions are separated proportional to the square root of the number of passes (Equation 1; Figure 2). With this increase in the length of path, researchers are able to gain a better separation of isobar pairs, positional isomers, and anomers (de Bruin et al., 2023; Leontyev et al., 2024; Ujma et al., 2019).

Using this technology, we analyzed neuronal subspecies of cardiolipins in primary neuronal culture and in mouse and pig neuronal tissues; cardiolipin composition profiles were created for basal conditions. With a single pass, cIMS-MS paired with the time-of-flight (TOF) analyzer was able to identify and isolate cardiolipin and its precursor, monolysocardiolipin, based on the charge state from other, more abundant phospholipids across all biological samples. Furthermore, using the multiple-pass capability of cIMS-MS, isolation and relative amplification of less abundant cardiolipin subspecies was achieved. With this improved resolution, researchers investigating cardiolipin as a potential therapeutic target will be able to determine how pharmacological interventions impact cardiolipin’s chain length and degree of saturation. This manuscript outlines the sample preparation and biological implications of using cIMS-MS to address the complexity of cardiolipin species.

## Methods

### Animals

All experimental procedures were performed in accordance with institutional guidelines and approved by the Institutional Animal Care and Use Committee under protocol #IACUC PRO00010498 and PRO00010274 at the University of Michigan. Yorkshire/Hampshire P6–8 piglets were purchased from Michigan State University. WT mice (C57BL/6J) were purchased from Jackson Laboratories. Floxed TAZ (exons 9 and 10) mice were provided by Dr. Douglas Strathdee (Ren et al., 2019). Rederived mice were backcrossed to a C57BL/6 background and maintained in accordance with University and NIH guidelines. Taz^*fl/fl*^ mice were crossed with Cg–Tg (Thy1-YFP-H; #003782) (Jackson Laboratories) to produce a Taz^*fl/fl*^–Thy1^*Cre*^ non-inducible knockout (KO) line.

### Primary cortical neuron isolation and culture

Primary cortical neurons were isolated from mouse brains as previously described (Anzell et al., 2021; Fogo et al., 2024). In brief, brains with cortical hemispheres were isolated on postnatal day 0–2 (P0–2). The tissue was digested with an enzymatic digestion solution [1x Hibernate complete media, 0.06 mg/mL L-cysteine (Sigma, 778,672), 1.4 × 10-4 N NaOH (sigma, 43,617), 10 nb/mL APV (2-amino-5-phosphonopentanoic acid, Sigma A-5282), and 50 µL of papain (Worthington, LS 03126)] and incubated at 37°C for 30 min. The tissue was then washed with Dulbecco’s phosphate buffered saline (DPBS) twice and replaced with Hibernate complete media. A cell suspension was created through pipetting up and down to dissociate the tissue. Cells were cultured in 60-mm dishes, coated with 0.01% polyethyleneimine (PEI), with a density of five million cells for 30 min at 37°C in 5% CO_2_. After 30 min, media was replaced with neurobasal complete media [1x Neurobasal Plus medium (Gibco, A3582901), 1% B27 plus (Gibco A365401), 0.5 mM GlutaMAX supplement (Gibco, 35050061), and 1% Penn/Strep (Gibco, SV30010)] and cultured at 37°C in 5% CO_2_ for 14 days with half media changes occurring every 3–4 days.

### Sample collection

#### Tissue collection

P6–8 male piglets were euthanized with Euthasol (150 mg/kg), and a transcardial perfusion with ice cold 1xDPBS was performed prior to sample collection. P10 mice were euthanized via isoflurane overdose, and samples were directly taken, with no perfusion required. Entire hemispheres from the mice were taken, while 5-g cortical and hippocampal biopsies were taken from the piglets. The tissue was immediately put on ice-cold PBS (1x). Tissue was weighed, and homogenization buffer A [10 mM HEPES (pH 7.5) (Sigma, H4034), 1 mM EDTA (Sigma, E6758), imM EGTA (Sigma, 03,777), 100 mM KCl (Sigma, P9333), 210 mM mannitol (Sigma, M4125), 70 mM sucrose (Sigma, S0389), and 1xHalt™ protease and phosphatase inhibitor cocktail (ThermoFisher, 784400)] was added (in ml) 10X to the weight (in grams) of the tissue. Mitochondrial fractionation was immediately performed (see below).

#### Isolated primary neuron sample preparation

Neurons were isolated and cultured for 14 days, as outlined above. Five million cells were plated on a 60-mm dish with media changes occurring every 3–4 days (NeuroBasal Plus Complete Media, Thermo Fisher Scientific). After 14 days, neurons were washed with cold 1x PBS before being suspended in 120 µL of homogenization buffer A, followed by mitochondrial fractionation (see below).

### Mitochondrial fractionation

This mitochondrial fractionation protocol was performed as previously described (Anzell et al., 2021; Sanderson et al., 2015). In brief, either brain tissue or primary neurons were collected using homogenization buffer A and homogenized using a Teflon Potter–Elvehjem homogenizer for 30 strokes at 300 rpm, resting the sample on ice every 10 strokes. The homogenates were collected in Eppendorf tubes (1.5 mL) and spun at 700 × g for 10 min at 4°C. After this first spin, the supernatant was collected and placed in a second Eppendorf tube (1.5 mL), while the pellet was discarded. The supernatant was spun again at 13,500 × g for 15 min. After this second spin, the supernatant was collected as the cytosolic fraction, while the pellet left in the tube was the mitochondrial pellet. The supernatant was decanted, leaving the mitochondrial pellet undisturbed in the tube. Once the mitochondrial pellet was isolated, lipid extraction or Western blots were performed (see below), and the remainder of the sample was stored at −80°C.

### Brain whole-cell lysates

Cortical mouse (0.1–0.5 g) tissue was weighed out and suspended in 1 mL of homogenization buffer A in a 2-mL Eppendorf tube, with an anticipated 500 µg of protein for every 100 mg of tissue. A sterile metal homogenization bead (Millipore Sigma BMSD113230TP) was placed in the tube (one bead per tube), and samples were shaken for 5 min at an oscillation frequency of 50 Hz (Qiagen Tissue Lyser LT 85600), followed by sonication. The protein concentration was measured using the Bradford Plus Assay (ThermoFisher, P123236), and samples were stored at −80°C.

### Western blots

Polyacrylamide gels (4%–20%, Invitrogen XP04205BOX) were loaded with 8–12 µg of the mitochondrial protein or whole-cell lysates and were transferred to nitrocellulose membranes. Membranes were then incubated with primary antibodies [1:500 anti-tafazzin (Santa Cruz sc-365810); 1:1000 anti-HSP60 (BD Transduction 611,562)] at 4°C overnight on a rocker. Membranes were washed thrice for 5 min each with Tris-Buffered saline and 0.1% Tween (TBST, Fisher Scientific, BP337500) and incubated in secondary antibodies [1:5000 donkey anti-mouse (Jackson Immuno Research Labs, 715-035-150) or 1:10,000 donkey anti-rabbit (Jackson Immuno Research Labs, 711-035-152)] for 60 min at room temperature. All antibodies were prepared with 2% BSA in TBST unless otherwise specified. Membranes were then washed thrice for 5 min each in TBST. Membranes were incubated in SuperSignalS West Pico PLUS chemiluminescent Substrate (Thermo Fisher, 34,577), imaged using an iBright 1500 imager (Invitrogen), and quantified by densitometry using FIJI.

### Lipid extraction

Mitochondrial fractionation was performed as described previously, and lipid extraction was performed using a combination of the Folch method and the Bligh and Dyer method, both of which are widely used in the field for lipid extraction from biological samples (Breil et al., 2017). In brief, 50 µL of deionized water was added to the mitochondrial pellets, followed by 150 µL of a 2:1 chloroform: methanol mix. Samples were vortexed for 15 s to resuspend the mitochondrial pellet. Samples were then spun at 17,000 × g for 1 min. After spinning, the samples were separated into three layers: the organic layer (bottom), protein pellet (middle), and aqueous layer (top). Using a pipette, the bottom organic layer was extracted and placed in a glass screw top vial (Fisher Scientific: 13,622,353). The samples were dried using 100% pure nitrogen and stored at −80°C.

### Lipid resuspension

For resuspension, 50 µL of pure 100% isopropanol alcohol (IPA) was added to the samples and vortexed. A measure of 5 µL of the sample was loaded into capillary tips and then ionized using the NanoLockSpray source operated in the negative mode at 1.0–1.3 kV.

### Capillary pipettes

Borosilicate capillary pipette tips, with filament, (Warner Instruments - Model No. G100TF-4) were pulled to a tip inner diameter of 5–10 µM using a P97 micropipette puller (Sutter, Navato, CA; Program #11) and coated in gold using an SC7620 Mini Sputter Coater (Quorum Technologies, Laughton, United Kingdom).

## Cyclic ion mobility mass spectrometry

All experiments were performed using a Select Series Cyclic Ion Mobility–Mass Spectrometer operated in the negative mode. (Waters Co. Milford, MA) (Giles et al., 2019). General settings were previously described but will briefly be discussed with an emphasis on cardiolipin-specific settings (Makey et al., 2024). The backing pressure was 2.35 mbar with a source pressure of 8.56 × 10^-3^. Trap traveling-wave ion guide was set to a pressure of 3.20 × 10^-2^ mbar of N_2_ gas with a helium cell flow rate of 120 mL/min at 2.05 mbar. The cyclic separation region was set to a rate of 40 mL/min of N_2_ at 1.76 mbar. The transfer cell pressure was set to 3.20 × 10^-2^ mbar of N_2_ gas. The TOF mass detector was set to V-mode (50–2000 m*/z*) with a pressure of 4.54 × 10^-7^. Traveling waves at 30 V wave height traveling at 1000 m/s were used to achieve ion mobility separation. Using 70 V and a repeller voltage of 100 V, the racetrack bias was set. Ion mobility data were collected over 200 bins at a rate of two pushes per bin, with a separation time of 2 ms and a resolution of 30,000–42,000. All data were analyzed using the software package MassLynx™ 4.2 (Waters Corp).

### Percent composition quantification

Due to the complexity of cardiolipin (isomers, isobars, and tissue specificity), relative quantification is often used. Cardiolipin and monolysocardiolipin are often compared as a ratio of the abundance of each species. This study uses the area under the curve of the chromatogram to determine parts of a whole of the cardiolipin content. To quantify the observed shifts in the cardiolipin content, cIM-MS data were recorded for 3 min for each sample, and the region of interest was extracted (Figure 4) and exported to MassLynx, retaining the drift time. The spectrum of each subspecies was exported as a chromatogram, and MassLynx was used to calculate areas under the curve for each subspecies. These areas were summed (total area) and divided by the area of each subspecies to determine the percent contribution of each species. UniDec software was used as a secondary source of composition validation (Marty et al., 2015)(Supplementary Figure S1). This provided an estimate of the relative amounts of cardiolipin and monolysocardiolipin in our samples as parts of a whole.

### Standards

Cardiolipin identification was confirmed using standards, as well as cross-referencing previous literature studies (Anthonymuthu et al., 2019a; Anthonymuthu et al., 2019b; Amoscato et al., 2014; Cole et al., 2018). All standards ordered from Avanti Polar Lipids, Inc. Standards that were delivered in powder form were resuspended in 100 uL of chloroform according to the manufacturer’s instructions.

16:0 Cardiolipin, 25 mg (Cat No: 710,333).

18:0 Cardiolipin, 25 mg (Cat No: 710,334).

14:0 Cardiolipin, 25 mg (Cat No: 750,332).

16:1 Cardiolipin, 5 mg (Cat No: 710,339).

18:1 Cardiolipin, 25 mg (Cat No: 710,335).

14:1 Cardiolipin, 5 mg (Cat No: 710,337).

16:0–18:1 Cardiolipin, 25 mg (Cat No: 710,341).

Cardiolipin internal standard mixture 1 (Cat No: LM6003).

### Statistics

Statistical analyses were performed using GraphPad Prism 8 (GraphPad Software, San Diego, CA). For comparisons between two different cardiolipin composition profiles (MLCL v Sat CL v Unsat CL), two-way ANOVAs were performed, with post hoc comparisons conducted using Sidak’s test, corrected for multiple comparisons. Unpaired, two-tailed t-tests were performed to assess expression changes between two different genotypes (TazThy1 v WT). For comparisons between cardiolipin subspecies composition between biological species, mixed-effect statistics were performed, with post hoc comparisons done between each biological species using Tukey’s test. Tests with p < 0.05 were considered statistically significant. For all experiments, *n* denotes experimental replicates. *A priori* power analysis determined four biological replicates for animal samples and three biological replicates for *in vitro* models.

## Results

### Verifying multiple pass in synthetic standards

To validate that cIMS-MS can identify cardiolipin, a variety of standards were analyzed, specifically the cardiolipin internal standard mix that contains several subspecies of cardiolipin [14:1 (3)–15:1 CL, 15:0 (3)–16:1 CL, 22:1 (3)–14:1 CL, 24:1 (3)–14:1 CL]. Using this standard mix, machine parameters were first optimized for cardiolipin. To further expand on the novelty of this method, we used the multiple-pass capability of cIMS-MS. When compared to traditional IMS-MS, cIMS-MS uses a circular separating path (Figures 2A,B). This allows for the selection of subspecies of interest and their subsequent multiple passes. The subspecies 22:1 (3)–14:1 CL and 24:1 (3)–14:1 are biologically relevant and similar in structure, size, and m/z. Using multiple-pass analysis with cIMS-MS, 22:1 (3)–14:1 and 24:1–14:1 (3) were isolated (Figure 2C) and subjected to multiple passes to achieve greater separation based on drift time on the chromatogram (Figure 2D). These two subspecies differ by six carbons (∼36 m/z due to the double charge) on the spectrum; however, within the chromogram, their drift time is so proximal that it is disguised as a single peak (Figure 2D). With additional passes, the chromatogram is able to separate the two subspecies into two distinct peaks (Figure 2D). This type of selectivity and isolation of cardiolipin subspecies provides proof-of-concept that multiple-pass cIMS-MS can be used to attain separation of CL subspecies in biological samples.

### Mitochondrial fractionation and lipid extraction

**FIGURE 3:**
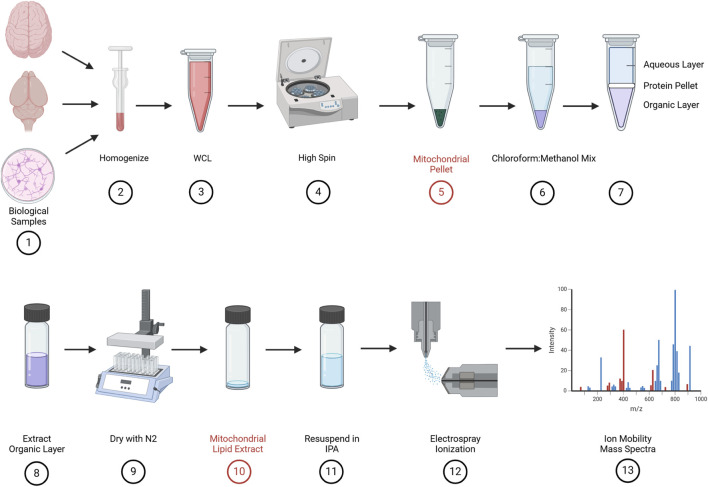
Biological sample preparation: samples were collected either from tissue (pig and mouse pup) or cell culture (primary cortical neurons) (#1) and suspended in ice-cold homogenization buffer A and homogenized (#2). Whole-cell lysates (WCLs) were collected after homogenization (#3), and a series of high spins (#4) results in a crude mitochondrial pellet (#5). The mitochondrial pellet was suspended in a 2:1 chloroform/methanol mix (#6), vortexed, and spun at 17 g. This produced three layers, namely, the aqueous layer on the top, a thin protein pellet in the middle, and the organic layer on the bottom (#7). The bottom, organic layer is carefully extracted and placed into a glass, screw top vial (#8) and dried with 100% pure nitrogen (#9). This produces a dried mitochondrial lipid extract (#10).

Traditional analysis of cardiolipin from biological samples with MS can result in cardiolipin being masked by more abundant lipids in the cell such as PG, PE, PC, and cholesterol. This makes detection, isolation, and quantification of cardiolipin difficult in whole-cell and tissue samples without additional separation methodologies. Mitochondrial isolation and fractionation were performed, resulting in a crude mitochondrial pellet, followed by lipid extraction (Figure 3). Cardiolipin makes up 15%–20% of the total lipid content in the inner mitochondrial membrane (IMM); therefore, enriching the mitochondrial content prior to lipid extraction increases the yield of cardiolipin and more accurate identification via mass spectrometry. The sample is resuspended in 50–100 uL of IPA and vigorously vortexed. Once the sample is resuspended, 5–10 uL of the sample is loaded into a borosilicate capillary pipette tip and loaded into the instrument for nanospray desorption electrospray ionization (Figure 3).

### Isolation based on the charge state of cardiolipin and monolysocardiolipin in mouse cortical tissue

**FIGURE 4:**
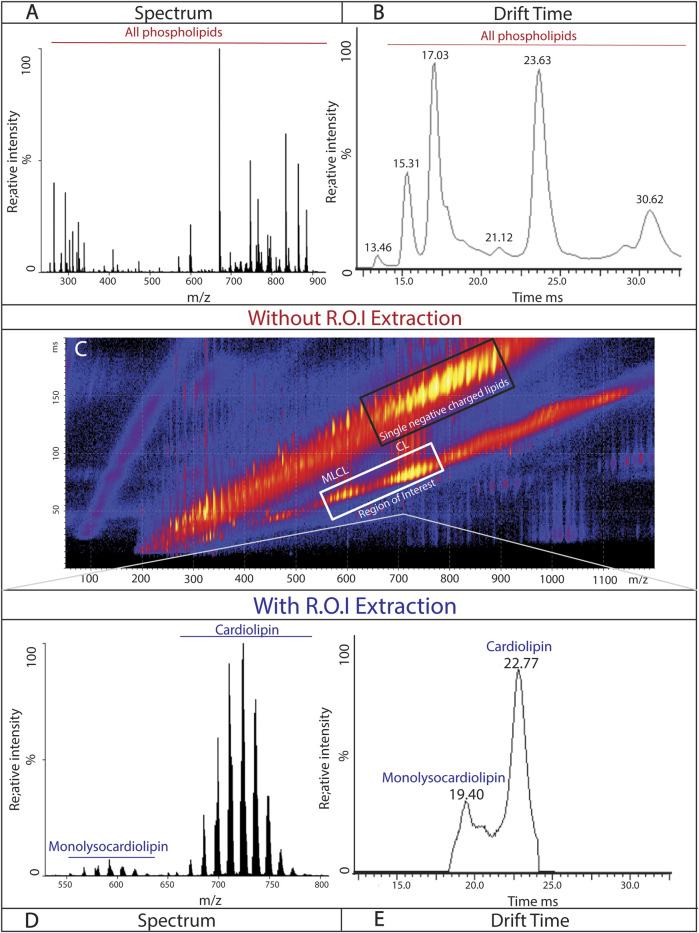
Isolation of cardiolipin and monolysocardiolipin based on the charge state: representative spectrum **(A)** and chromatogram **(B)** of phospholipids (doubly and singly negatively charged) from the isolated mitochondria of mouse cortical tissue. **(C)** Continuous spray of mitochondrial lipids isolated from mouse cortical tissue, demonstrating the separation of singly negative-charged lipids (black box; top) and doubly negative-charged lipids (white box; bottom; region of interest). Representative spectrum **(D)** and chromatogram **(C)** from mouse cortical tissue of cardiolipin and monolysocardiolipin after extraction of region of interest.

Phospholipids involved in the membrane structure (PG, PC, PE, and PA) have a single negative charge, while cardiolipin and monolysocardiolipin carry double negative charges. Without separation based on the charge state, cardiolipin and monolysocardiolipin are masked by the more abundant, singly charged phospholipids (Figures 4A,B). However, with a single pass around the circular “racetrack” that characterizes cIMS-MS, ions are separated based on their single or double negative charges. This separation allows for the identification and selection of double negative charged species, such as cardiolipin and monolysocardiolipin, while excluding other, more abundant phospholipids from analysis (Figure 4C). Region of interest (ROI) (i.e., cardiolipin and monolysocardiolipin) was selected (Figure 4C, white box) and extracted, while retaining drift time, for analysis via MassLynx™. The charge-based separation coupled with the ROI selection method results in an accurate spectrum and chromatogram of cardiolipin and monolysocardiolipin (Figures 4D,E). Differences in the mass/charge ratio between cardiolipin and monolysocardiolipin also separate cardiolipin from its precursor, which only differs in a drift time of 3.3 ms (Figures 4D,E; Supplementary Figure S2), with a single pass on the cIMS-MS. The reproducibility of separation based on the charge state and ROI extraction was tested in cortical tissue extracted from mouse pups with drift time replication and peak width compared across samples and technical replicates (Supplementary Figure S3).

**FIGURE 5:**
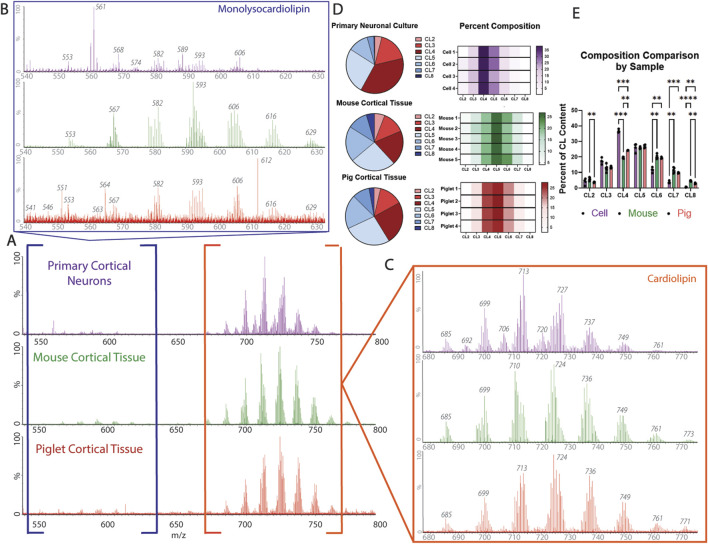
Cardiolipin profile composition across biological samples: **(A)** representative spectrum from the primary neuron culture (top; purple), mouse cortical tissue (middle; green), and pig cortical tissue (bottom; red). **(B)** Monolysocardiolipin extraction from the spectrum of each biological sample [primary neuronal culture (top; purple), mouse cortical tissue (middle; green), and pig cortical tissue (bottom; red)]. **(C)** Cardiolipin extraction from the spectrum of each biological sample [primary neuronal culture (top; purple), mouse cortical tissue (middle; green), and pig cortical tissue (bottom; red)]. Identification of main cardiolipin and monolysocardiolipin subspecies, degree of saturation, and acyl chain make up of interest are identified in Table 1. **(D)** Cardiolipin profile broken down by subspecies across each biological sample (*n* = 4-5). **(E)** Composition comparison of cardiolipin subspecies between biological samples, demonstrating that the primary neuronal culture has the most significant difference in the cardiolipin profile compared to mouse and pig cortical tissues (CL4, CL6, CL7, and CL8), whereas the cardiolipin profile composition is only significantly different between mouse and pig cortical tissues in subspecies CL2, CL4, and CL8 (*n* = 4-5; mixed-effect analysis, * = p < 0.05, ** = p < 0.01, and *** = p < 0.001).

To expand on the utility of this method, the ability of cIMS-MS to separate cardiolipin and monolysocardiolipin based on the charge state was tested in additional biological samples from neural tissues commonly used to model acute and chronic neurologic diseases (Figure 5: (1) primary cortical neurons, commonly used for *in vitro* disease modeling; (2) mouse cortical brain tissue, for transgenic and rodent models of disease; (3) pig brain tissue, for large animal and translational applications (Figures 5A–C). Monolysocardiolipin and cardiolipin subspecies were identified, and the composition of the cardiolipin profile under basal conditions across all experimental systems was established, demonstrating the broad and translational applicability of using cIMS-MS for cardiolipin evaluation (Figure 5D). Using both MassLynx software and Unidec software, the composition of cardiolipin subspecies was determined as relative percentages in reference to the most prominent subspecies (Supplementary Figure S1). From this analysis, the composition of the cardiolipin content was assessed and compared across each model, establishing a baseline profile in several biological systems (Figure 5E).

**TABLE 1:** Using synthetic standards, Unidec Software, and cross-referencing the literature, identification of main cardiolipin and monolysocardiolipin subspecies, theoretical molecular weight, degree of saturation, and acyl composition.

	Monolysocardiolipin			
	MLCL 1	MLCL 2	MLCL 3	MLCL 4
m/z	582	593	606	618
Theoretical molecular weight	1163	1185	1211	1235
Acyl carbons:double Bonds	(52:2)	(54:4)	(56:5)	(58:8)

	Cardiolipin			
	Premature, more saturation and shorter fatty acid tails			
	CL 1	CL 2	CL 3	CL 4
m/z	679	685	699	710
Theoretical molecular weight	1357	1369	1397	1419
Acyl carbons: double bonds	(66:2)	(66:4)	(68:4)	(70:5)

	Cardiolipin			
	Mature, more unsaturation and longer fatty acid tails			
	CL 5	CL 6	CL 7	CL 8
m/z	724	736	749	761
Theoretical molecular weight	1447	1471	1497	1521
Acyl carbons:double bonds	(72:8)	(74:10)	(76:11)	(78:13)

### Genetic manipulation to validate the cardiolipin and monolysocardiolipin profiles

**FIGURE 6:**
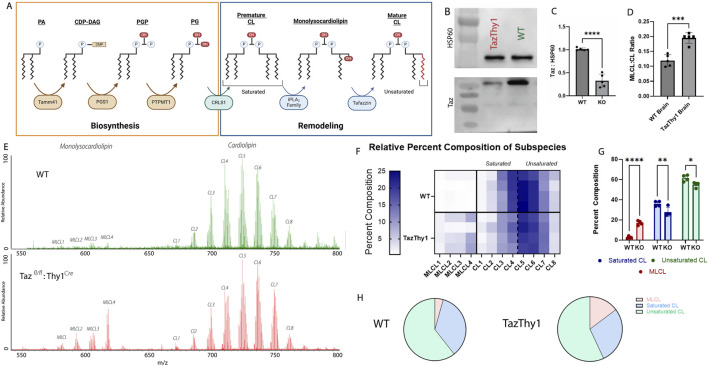
Genetic manipulation of cardiolipin remodeling. **(A)** The biosynthesis and remodeling pathway of cardiolipin. Cardiolipin biosynthesis begins with PA being imported into the matrix and is concluded with cardiolipin synthase 1 converting PG into premature cardiolipin. Then, the remodeling process begins with premature cardiolipin being converted into monolysocardiolipin by the iPLA_2_ family. Monolysocardiolipin is then converted into mature cardiolipin by the enzyme tafazzin. The main difference between premature and mature cardiolipin is the degree of saturation of the four acyl chains. **(B, C)** Quantification and representation of Western blots of whole-cell lysates from Taz^*fl/fl*^:Thy1^*Cre*^ p10 mice from the brain confirming knockout (*n* = 5; unpaired, two-tailed t-test; p < 0.05). **(D)** Relative quantification of the monolysocardiolipin:cardiolipin ratio from cortical tissue collected from the cortical regions of p10 WT and Taz^*fl/fl*^:Thy1^*Cre*^ mice (*n* = 5; unpaired, two-tailed t-test; p < 0.05). **(E)** Representative spectrum from the cortical regions of p10 WT (Top) and Taz^*fl/fl*^:Thy1^*Cre*^ (Bottom) mice. **(F)** Heatmap assessing the composition of major cardiolipin and monolysocardiolipin subspecies in wildtype and Taz^*fl/fl*^:Thy1^*Cre*^ p10 mice, demonstrating the accumulation of monolysocardiolipin. **(G)** Quantification of the composition of monolysocardiolipin, saturated cardiolipin, and unsaturated cardiolipin in wildtype and Taz^*fl/fl*^:Thy1^*Cre*^ p10 mice, demonstrating the depletion in cardiolipin content, primarily from saturated cardiolipin (premature cardiolipin) and accumulation of monolysocardiolipin validating cardiolipin and monolysocardiolipin identification through genetic manipulation (*n* = 5; two-way ANOVA, * = p < 0.05, ** = p < 0.01, and *** = p < 0.001). **(H)** Representative part of a whole from wildtype and Taz^*fl/fl*^:Thy1^*Cre*^ p10 mice.

The identification and quantification of cardiolipin and monolysocardiolipin is a valuable method for diagnosing Barth syndrome. Barth syndrome is a rare X-linked disease caused by dysfunctional mutations in the *Taz* gene, resulting in impaired cognition, stunted growth, and often fatal cardiomyopathy. The *Taz* gene encodes for the enzyme tafazzin, the main enzyme responsible for reacylating monolysocardiolipin to mature cardiolipin (Figure 6A). Under normal conditions, there is minimal monolysocardiolipin expression; however, when tafazzin cannot function properly, there is an accumulation of monolysocardiolipin. There is a clinical need to quickly and reliably separate and quantify cardiolipin and monolysocardiolipin levels for diagnosis.

To validate the accuracy of cIMS-MS as a method to quantify relative cardiolipin and monolysocardiolipin expressions, a neuronal-specific *Taz*-knockout mouse was optimized. Crossing a Taz^*fl/fl*^ mouse with the Thy1^*Cre*^ mouse generated mice with *Taz* knockout in neurons that was active at embryonic day 10. To assess whether the Taz^*fl/fl*^ × Thy1^*Cre*^ line was neuronal-specific, brain lysates were analyzed through Western blot for tafazzin protein expression, which resulted in significant knockout in the brain (Figures 6B–E). cIMS-MS was used to determine whether monolysocardiolipin accumulated in Taz KO mice to validate the specificity of cIMS-MS to identify these species. Cortical brain samples from postnatal day 10 mice were analyzed using cIMS-MS to quantify the ratio of monolysocardiolipin to cardiolipin levels. In the Taz^*fl/fl*^:Thy1^*Cre*^ samples, there was a significant decrease in tafazzin expression and a significant increase in monolysocardiolipin:cardiolipin, providing loss-of-function validation that cIMS-MS spectra are specific to MLCL (Figures 6B–E). This monolysocardiolipin accumulation mimics the accumulation observed in Barth syndrome patients lacking functional Taz expression (Valianpour et al., 2005). Furthermore, there was a depletion in both mature and premature total cardiolipin content, with a specific reduction in the saturated cardiolipin, which is consistent with pre-mature cardiolipin, as observed in other studies (Oemer et al., 2020) (Figures 6F–H). Through genetic manipulation of cardiolipin remodeling, these data confirm that the spectrum and chromatogram reflect cardiolipin and monolysocardiolipin. Overall, these data support the premise that cIMS-MS separation can be used to rapidly separate and produce relative quantitative data for cardiolipin and monolysocardiolipin present in biological samples. Future validation is required in clinical samples (i.e., human cells and blood samples); however, this quick and reliable method to assess the monolysocardiolipin:cardiolipin ratio may have significant clinical implications with additional validation.

### Multiple passes in biological samples

Cardiolipin has the ability to express tissue specificity, and the brain contains the highest variety of cardiolipin subspecies. Using the multipass capability of the cIMS-MS, less abundant cardiolipin subspecies were able to be isolated and signals amplified when compared to the other, more abundant subspecies. The most abundant cardiolipin subspecies in cortical samples from mouse and pig is CL5 [724 m*/z*, (72:8)], while the least abundant is CL8 [761 m*/z* (78:13)]. Using cIMS-MS multiple pass, the signal from CL8 was isolated from the spectrum and amplified.

The sample was introduced using TOF to establish a strong, stable signal before switching to the cyclic ion mobility mode. The peak of interest, CL8, was identified on the continuous drift time readout, determining the drift time of CL8 completing a single pass. The drift time was analyzed using a MassLynx pass calculator to determine the drift time for each number of passes for CL8.

**FIGURE 7:**
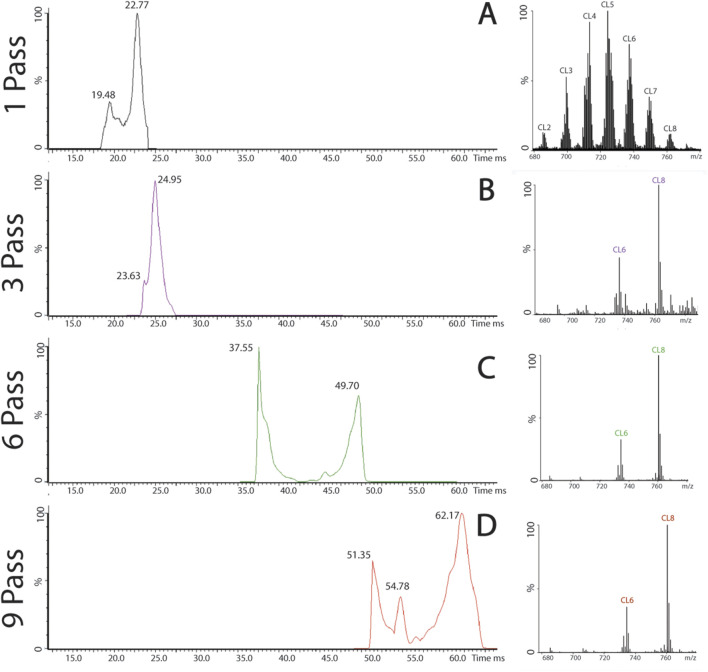
Multiple passes in pig cortical tissue. **(A)** Pig cortical tissue after a single pass, separated by charge state, cardiolipin chromatogram, and spectrum. **(B)** Isolation of CL8, chromatogram, and spectrum after three, six **(C)**, and nine passes **(D)**.

Using the pig cortex sample, nine passes were successfully completed to isolate the signal of CL8 on the spectrum and chromatogram (Figure 7). The isolation of CL8 was also achieved in the mouse brain sample (Supplementary Figure S4). As the number of passes increased, heavier subspecies began to “lap” the lighter subspecies, which can complicate analysis, a phenomenon commonly referred to as “wraparound” (Supplementary Figure S5). Resolution at the level of individual subspecies may expand the ability for researchers to better examine how different cardiolipin subspecies are altered throughout disease or injury pathology and its impact on the overall composition of the cardiolipin profile.

## Conclusion

Cardiolipin is essential for maintaining mitochondrial health and overall cell homeostasis. Recent studies have uncovered a critical role for cardiolipin in neurological disorders and brain injury pathologies, revealing the need for precise and accurate cardiolipin detection and analysis. Historically, cardiolipin was analyzed using several forms of mass spectrometry, mainly LC-MS (Bautista et al., 2022). This technique is widely used and accepted in the field and has produced detailed accounts of cardiolipin as a potential biomarker in traumatic brain injury, ischemia/reperfusion injury, tumor environments, and cardiac disease (Falabella et al., 2021; Dudek, 2017).

This study uses the novel method of cIMS-MS in the analysis of cardiolipin. This protocol will allow researchers to assess the cardiolipin content quickly and accurately from lipids extracted from a crude mitochondrial fraction. cIMS-MS also allows for the separation of cardiolipin from other, more abundant phospholipids based on the charge state (Figure 4). Using this separation, compositions of cardiolipin profiles were established and compared across several biological samples (Figure 5).

Furthermore, cIMS-MS was able to separate cardiolipin from its precursor, monolysocardiolipin, which is strikingly similar in structure, m/z, and drift time. Using a neuronal-specific knockout of Tafazzin, the enzyme that is required for the conversion of monolysocardiolipin to cardiolipin, monolysocardiolipin accumulation was observed, along with a significant decrease in the cardiolipin content, specifically in the saturated cardiolipin subspecies (Figures 6G,H). The ability to separate cardiolipin from monolysocardiolipin is clinically relevant as the monolysocardiolipin:cardiolipin ratio is a primary method for diagnosing Barth syndrome. Demonstrating monolysocardiolipin accumulation and cardiolipin depletion with genetic manipulation of cardiolipin remodeling provides additional validation of the authenticity of monolysocardiolipin and cardiolipin subspecies in the cardiolipin profile after cIMS-MS.

Additionally, using the multi-pass setting on the cIMS-MS, less abundant cardiolipin subspecies were able to be isolated and amplified. Validated in synthetic standards, CL8 (78:13), the least abundant cardiolipin subspecies across all biological samples, was isolated and amplified through various passes (Figures 2, 7, Supplementary Figures S4, 5). This technology will allow more in-depth investigation into cardiolipin profile changes in disease and injury pathologies. This could help researchers understand, from a mechanistic perspective, how cardiolipin is being remodeled following injury or in disease states. This technology is promising to assess not only cardiolipin but also the entire lipid profile in different injury pathologies or neurological diseases. This information will further our knowledge of mechanistic changes before and after injury and help guide researchers in designing potential therapeutics.

## Data Availability

The raw data supporting the conclusions of this article will be made available by the authors, without undue reservation.

## Supplementary material

Supplementary Figure S1
**(A)** Unidec software breaking down composition calculation, which was used to calculate the composition profiles for each biological sample (Marty et al., 2015).

Supplementary Figure S2 Separation of cardiolipin and monolysocardiolipin despite close drift time. **(A)** Representative spectrum from primary cortical neurons of isolated monolysocardiolipin (top; 550–630 m/z) and cardiolipin (bottom; 670–800 m/z). **(B)** Chromatogram of the drift time of monolysocardiolipin (top; 19.33) and cardiolipin (bottom; 22.77), demonstrating a difference in the drift time of 3.44 ms.

Supplementary Figure S3 Technical reproducibility. **(A)** Stack of cardiolipin drift time from tissue isolated from four different mouse cortical hemispheres (four biological replicates), demonstrating the reproducibility of a cardiolipin chromatogram and spectrum. **(B)** A representative cardiolipin spectrum from one of the above mouse cortical tissue samples. **(C)** Magnification of the most prevalent cardiolipin subspecies in the mouse cortical tissue (CL5; 724 m/z) with its peak width (FWHM). **(D–E)** Technical replicates of the same cortical mouse tissue sample (top) and primary cortical neuronal sample (bottom) ran on separate days with different capillary pipettes to demonstrate the reproducibility.

Supplementary Figure S4 Multiple passes in mouse cortical tissue. **(A)** Mouse cortical tissue after a single pass, separated by charge state, cardiolipin chromatogram, and spectrum. **(B)** Isolation of CL8, chromatogram, and spectrum after three, six **(C)**, and nine passes **(D).**

Supplementary Figure S5 Multiple passes with overlapping subspecies in pig hippocampus. **(A)** Pig hippocampal tissue after a single pass, separated by charge state, cardiolipin chromatogram, and spectrum. **(B)** Isolation of CL8, chromatogram, and spectrum after three and six passes **(C). (D)** At nine passes, the chromatogram shifts back to one massive peak, suggesting that species are beginning to “lap each other,” a phenomenon referred to as “wraparound.”
