# Supplementary figures and images for: Analysis of neuronal cardiolipin and monolysocardiolipin from biological samples with cyclic ion mobility mass spectrometry
Source: Front Physiol. 2025 May 29;16:1592008. doi: 10.3389/fphys.2025.1592008 (PMC12159609; doi:10.3389/fphys.2025.1592008)

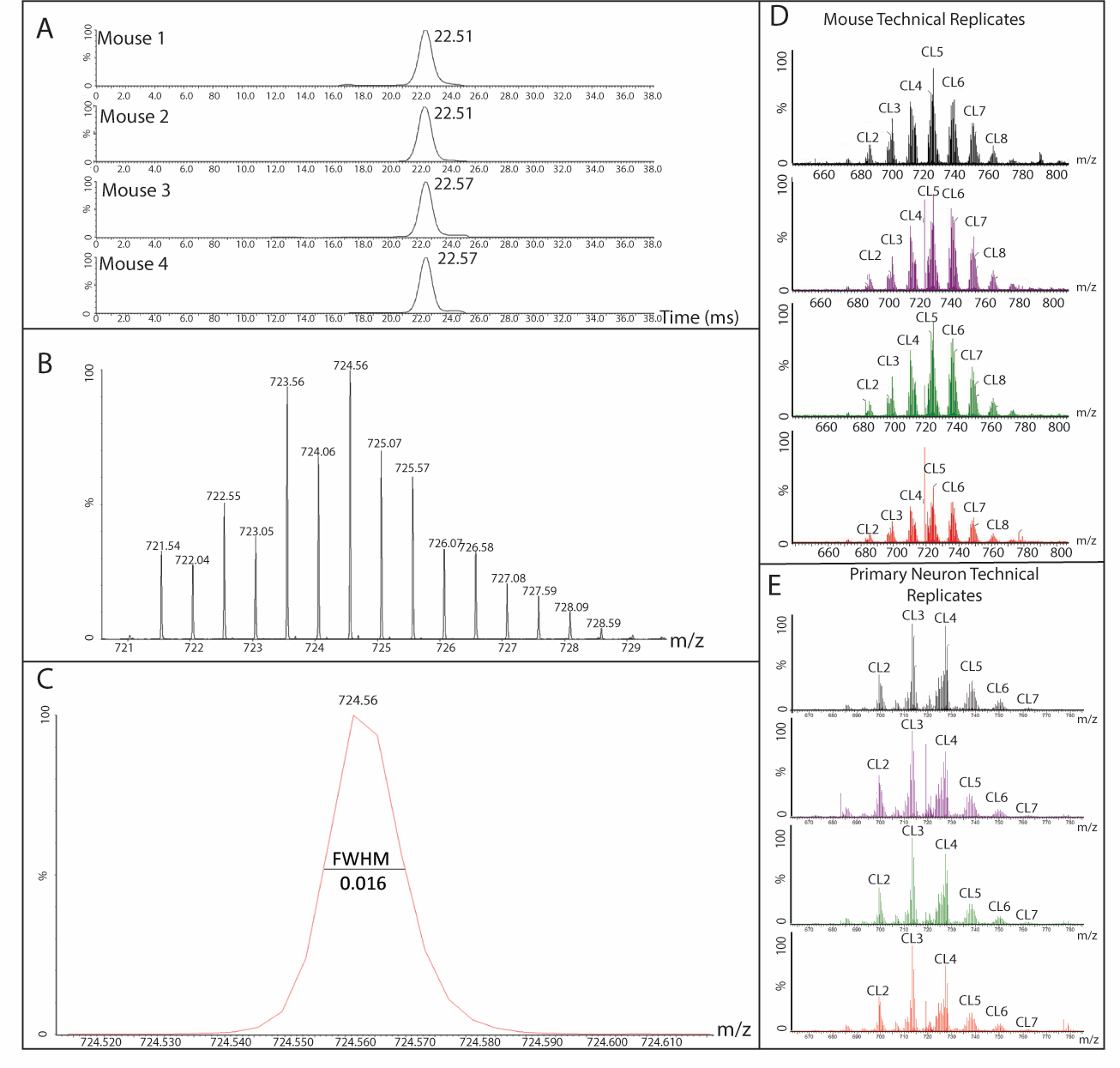

Supplement: Supplementary file 1 [file Image3.tif]

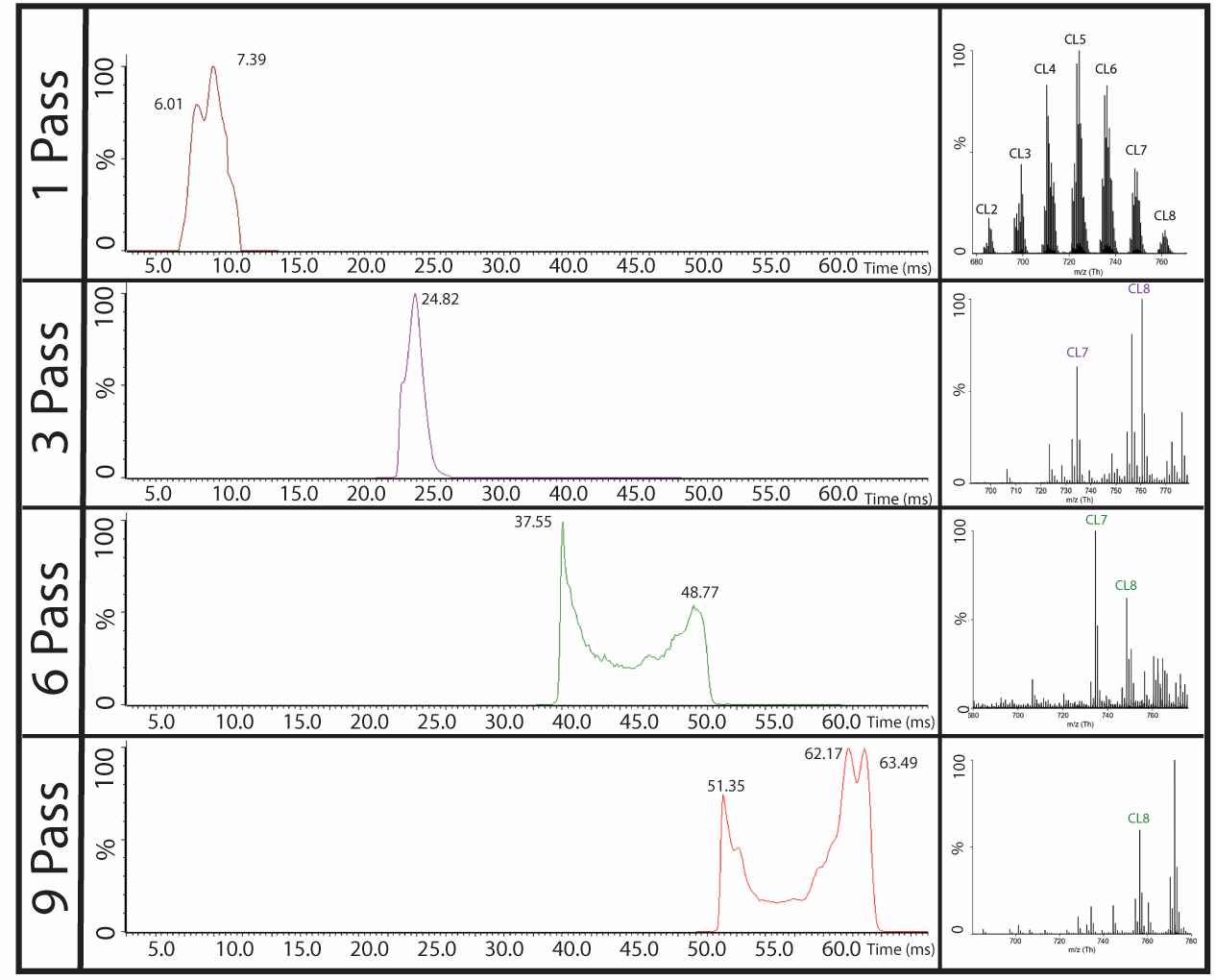

Supplement: Supplementary file 2 [file Image4.tif]

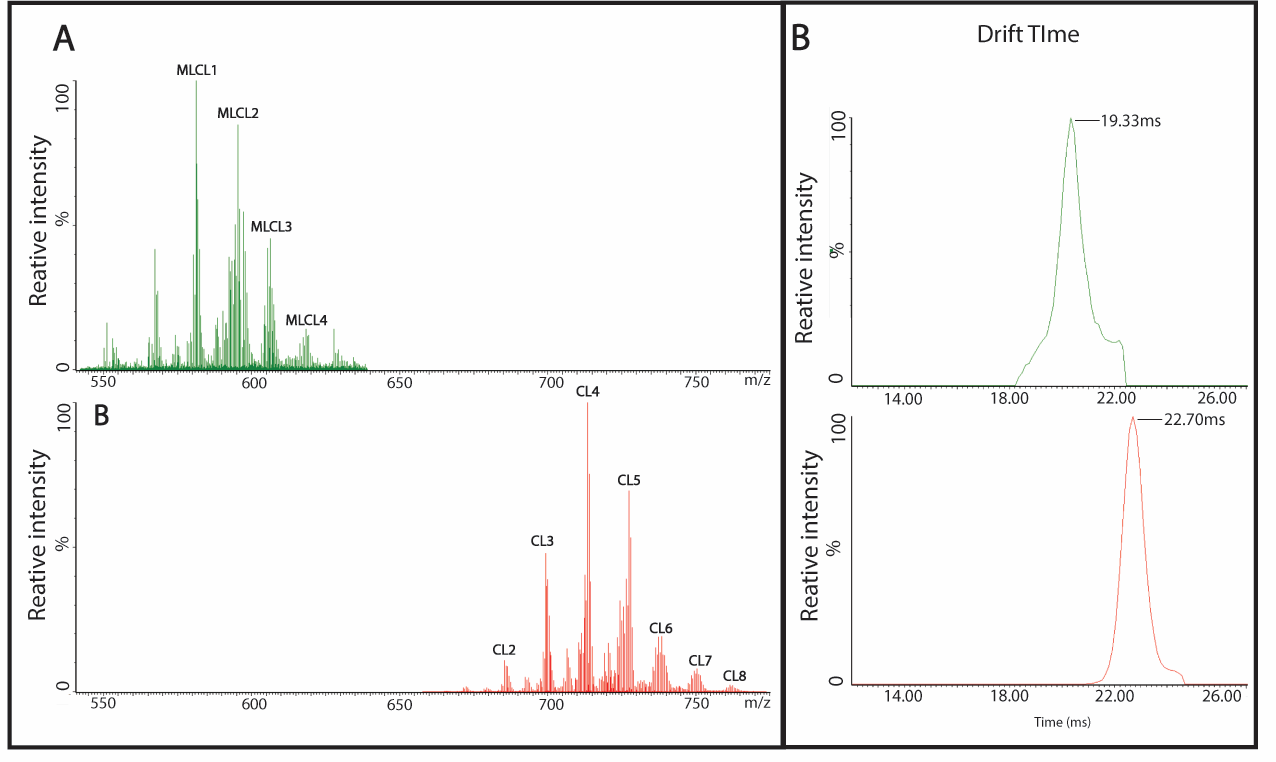

Supplement: Supplementary file 3 [file Image2.tif]

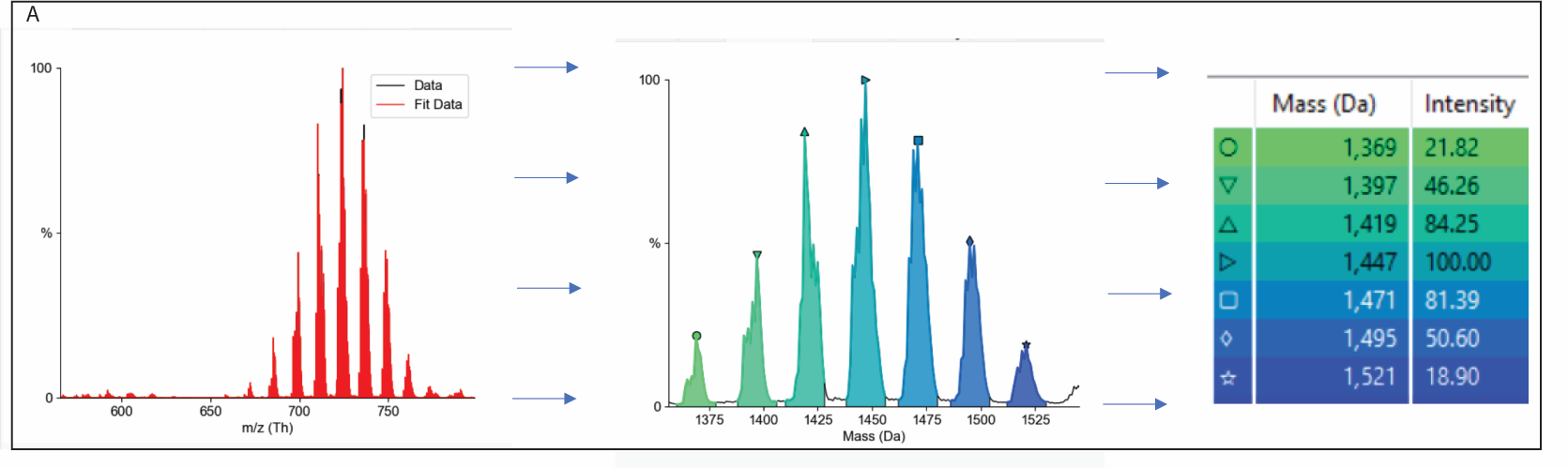

Supplement: Supplementary file 4 [file Image1.tif]

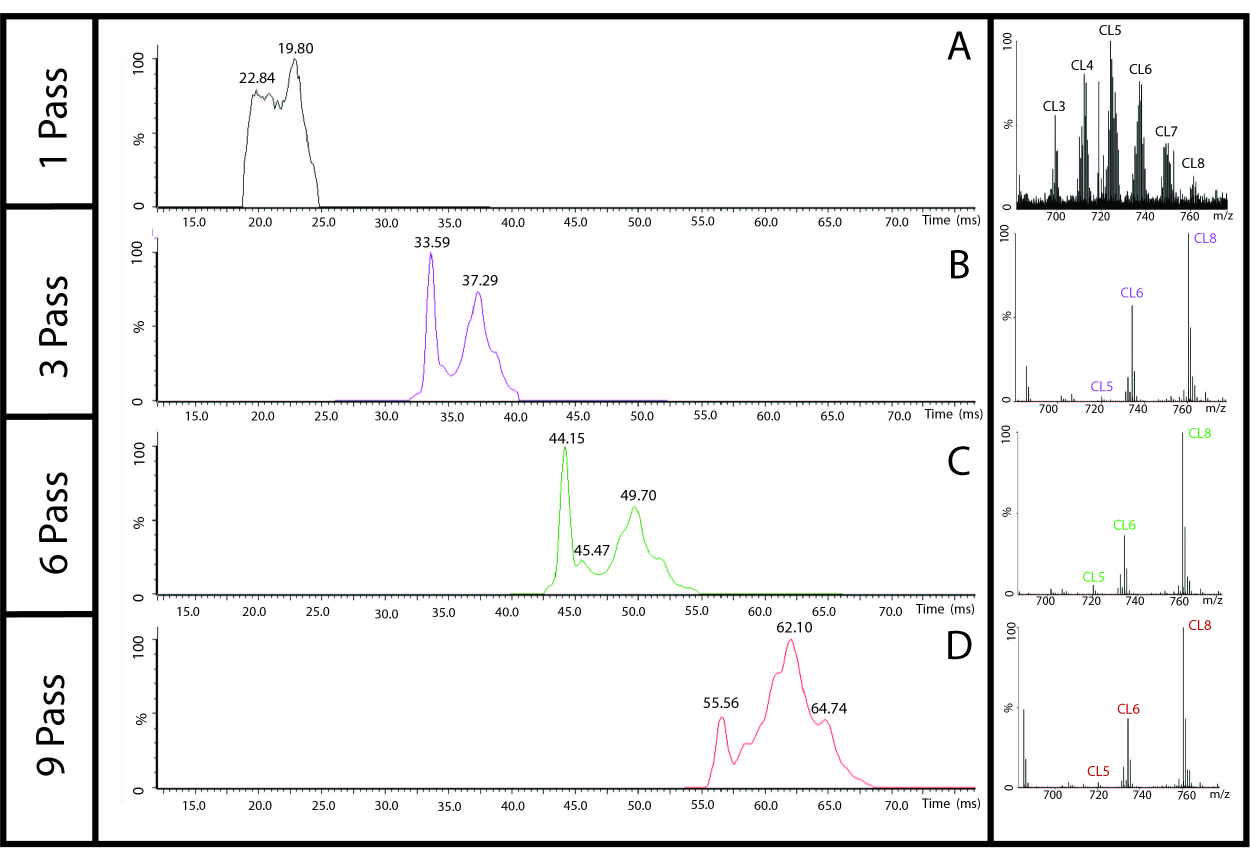

Supplement: Supplementary file 5 [file Image5.tif]
